# A novel *Atg5*-shRNA mouse model enables temporal control of Autophagy *in vivo*

**DOI:** 10.1080/15548627.2018.1458172

**Published:** 2018-07-12

**Authors:** Liam D. Cassidy, Andrew RJ. Young, Pedro A. Pérez-Mancera, Birgit Nimmervoll, Adil Jaulim, Hung-Chang Chen, Dominick J. O. McIntyre, Rebecca Brais, Thomas Ricketts, Simon Pacey, Maike De La Roche, Richard J. Gilbertson, David C. Rubinsztein, Masashi Narita

**Affiliations:** a Cancer Research UK Cambridge Institute, University of Cambridge, Cambridge, UK; b Department of Histopathology, Cambridge University Hospitals NHS Foundation Trust, Cambridge, UK; c Cambridge Institute for Medical Research, Department of Medical Genetics, Cambridge, UK; d Department of Oncology, University of Cambridge, Cambridge, UK; e UK Dementia Research Institute, Cambridge Biomedical Campus, Cambridge Biomedical Campus, Cambridge, UK

**Keywords:** ATG5, autophagy, fibrosis, genetically engineered mouse model, liver, shRNA

## Abstract

Macroautophagy/autophagy is an evolutionarily conserved catabolic pathway whose modulation has been linked to diverse disease states, including age-associated disorders. Conventional and conditional whole-body knockout mouse models of key autophagy genes display perinatal death and lethal neurotoxicity, respectively, limiting their applications for *in vivo* studies. Here, we have developed an inducible shRNA mouse model targeting *Atg5*, allowing us to dynamically inhibit autophagy *in vivo*, termed ATG5i mice. The lack of brain-associated shRNA expression in this model circumvents the lethal phenotypes associated with complete autophagy knockouts. We show that ATG5i mice recapitulate many of the previously described phenotypes of tissue-specific knockouts. While restoration of autophagy in the liver rescues hepatomegaly and other pathologies associated with autophagy deficiency, this coincides with the development of hepatic fibrosis. These results highlight the need to consider the potential side effects of systemic anti-autophagy therapies.

## Introduction

Macroautophagy (herein referred to as autophagy) is an evolutionarily conserved, bulk cellular degradation system required to maintain cellular and energy homeostasis. Through autophagy, cytoplasmic components such as lipids, proteins and entire organelles are isolated within double-membrane vesicles (autophagosomes) and subsequently delivered to the lysosome to facilitate degradation and recycling of their respective constitutive components. Deregulation of autophagic flux, the rate at which autophagosomes form, fuse with lysosomes, and break down constitutive components, is thought to play a key role in the development of age-associated disorders, neurodegenerative conditions, and cancer [1–7].

Conventional knockout mouse models of essential autophagy genes, such as *Atg5* and *Atg7*, display perinatal lethal phenotypes within the first day after birth. This coincides with a period of metabolic stress wherein neonates must adapt and engage the autophagic machinery to mobilise their own food stores [8,9]. Additionally, conditional whole-body knockout of *Atg7* in adult mice has provided further evidence for the requirement of autophagy to survive acute metabolic stress. These mice also experience premature death due to neurotoxicity in metabolically unstressed states [10].

Due to these limitations, there has been a reliance on tissue-specific knockouts to provide insight into the role of basal autophagy in tissue development and homeostasis [11]. However, in all such classical approaches there is a dependence on the complete and irreversible abrogation of key autophagy genes, a situation not generally associated with the etiology of human disease or therapeutic modulation of autophagy. These approaches preclude the ability to perform reversal experiments and restore autophagy as a mechanism to reverse or modulate the disease state.

With this in mind, we have generated an inducible *Atg5-*shRNA mouse model, which enables us to inhibit and restore ATG5-dependent autophagy *in vivo*. The model enables temporal control of *Atg5* levels, which can be made ubiquitous or cell type-specific through breeding with appropriate CRE-recombinase expressing strains. Herein we have chosen to focus on the characterization of systemic *Atg5* downregulation and provide evidence that these mice display key phenotypes analogous to those described in knockout models (e.g., hepatomegaly, reduced adipose tissue, and pancreatic degeneration), indicating a high degree of *Atg5* knockdown and autophagy inhibition in these tissues. Importantly, we further utilise the system to ascertain how reversible these pathological states are, and provide evidence that autophagy inhibition and subsequent restoration may have pathological consequences.

## Results

**Figure 1.**
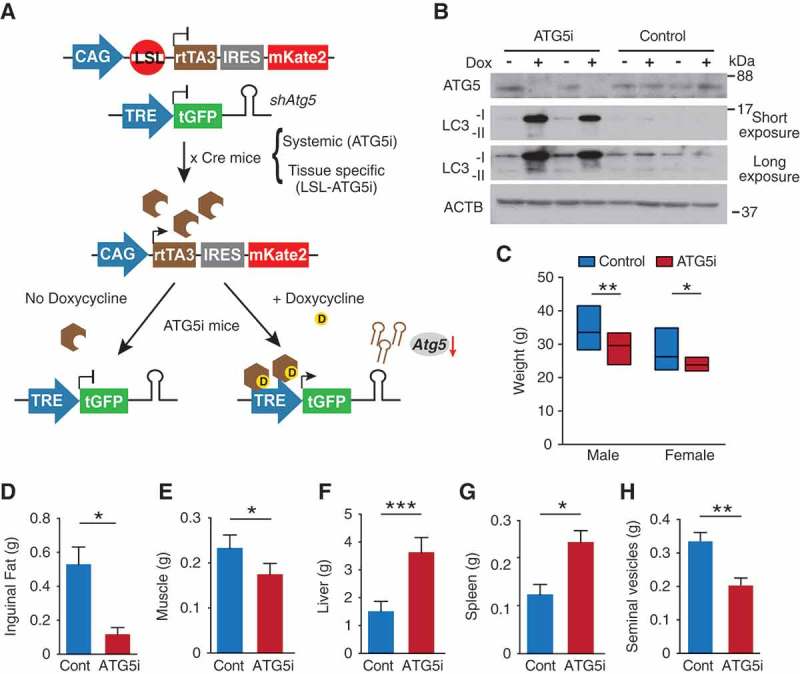
Generation of ATG5i mice. (a) Graphical illustration of doxycycline (dox)-inducible *Atg5*-RNAi (ATG5i) system. Only through crossing with the appropriate Cre-expressing strain is the loxP-STOP-loxP (LSL) cassette excised and rtTA3 protein produced. In the presence of dox, rtTA3 is able to bind to the tet-responsive element (*TRE*) and drive the expression of *Atg5*-shRNA in a miR-E backbone. IRES, Internal Ribosome Entry Site; mKate2, far-red fluorescent protein (Evrogen). (b) Western blots for the indicated proteins in MEFs isolated from ATG5i mice and littermate controls in the presence or absence of dox (3 days). Control littermates lack either the rtTA3 or sh*Atg5* cassette. (c-h) Eight-week-old ATG5i mice fed on a dox-containing diet for 6 weeks display a decrease in (c) weight (males, P = 0.0017 n = 16 control and ATG5i; females, P = 0.0239 n = 16 control and 9 ATG5i mice), (d) reduction in inguinal fat weight (P = 0.0286, n = 4 males per condition), (e) reduction in muscle weight (P = 0.0286, n = 4 males per condition), (f) hepatomegaly (P = 0.0006, n = 7; 3 females and 4 males per condition), (g) splenomegaly (P = 0.0286 n = 4 males per condition), and (h) seminal vesicle atrophy (P = 0.0022 n = 6 males per condition). All pairwise comparisons determined using Mann-Whitney test (*P < 0.05, **P < 0.01 and ***P < 0.001). Error bars represent s.d. around the means.

To investigate the effects that a reduction in autophagy would have on organismal homeostasis, and the degree to which these effects are reversible, we have developed a mouse model incorporating a doxycycline (dox)-inducible shRNA system (Figure 1(a)) [12,13]. Using a sensor-based screening system [14], we first obtained a panel of shRNAs against *Atg5*, encoding an essential component for autophagosome formation, and then selected an shRNA with the greatest knockdown *in vitro, Atg5_1065* (#1 in Fig. S1, see Methods). Subsequently, mice were generated carrying a single copy of *Atg5_1065* under the control of doxycycline (dox). Briefly, the TRE (tetracycline-responsive element)-regulated *Atg5*-shRNA is downstream of the *Col1a1* locus and is GFP-linked providing a non-invasive reporter system of activation. The shRNA transcription is driven in mice by the transgene rtTA3 in the presence of dox, of which spatial expression is restricted courtesy of a loxP-stop-loxP (LSL) cassette (this model is termed LSL-ATG5i mice). To generate a second version of the mouse model, wherein *Atg5* can be ubiquitously knocked down, we crossed the LSL-ATG5i mouse to a *Pgk1-Cre*-expressing strain [15]. This resulted in germ-line excision of the LSL ‘STOP cassette’, which was passed on to subsequent generations, while the *Pgk1-Cre* was rapidly bred out (ATG5i mice). ATG5i mouse embryonic fibroblasts (MEFs) show a reduction in ATG5 levels and the conversion of soluble LC3-I to membrane bound LC3-II, an ATG5-dependent process, by western blot analysis upon administration of dox (Figure 1(b)).

Using a tamoxifen-inducible Cre (Cre-ERT2) system, *Atg7* was recently knocked out systemically in adult mice [10]. To test the extent to which ATG5i mice recapitulate gross phenotypes of the whole-body somatic *atg7* knockout (KO) mice, 8-week old ATG5i mice were placed on a dox-containing diet for 6 weeks. Similar to the systemic *atg7* KO mice, ATG5i mice on dox appeared smaller in size with smaller weight gain in both genders (Figure 1(c)). Anatomical inspection revealed, as in the whole-body somatic *atg7* KO mice and/or *atg5* and *atg7* KO mice, a reduction of fat [16] and muscle tissues (Figures 1(d,e)) [17], with the presence of hepatomegaly [9], splenomegaly, and seminal vesicle atrophy (Figure 1(fh)) [18].

**Figure 2.**
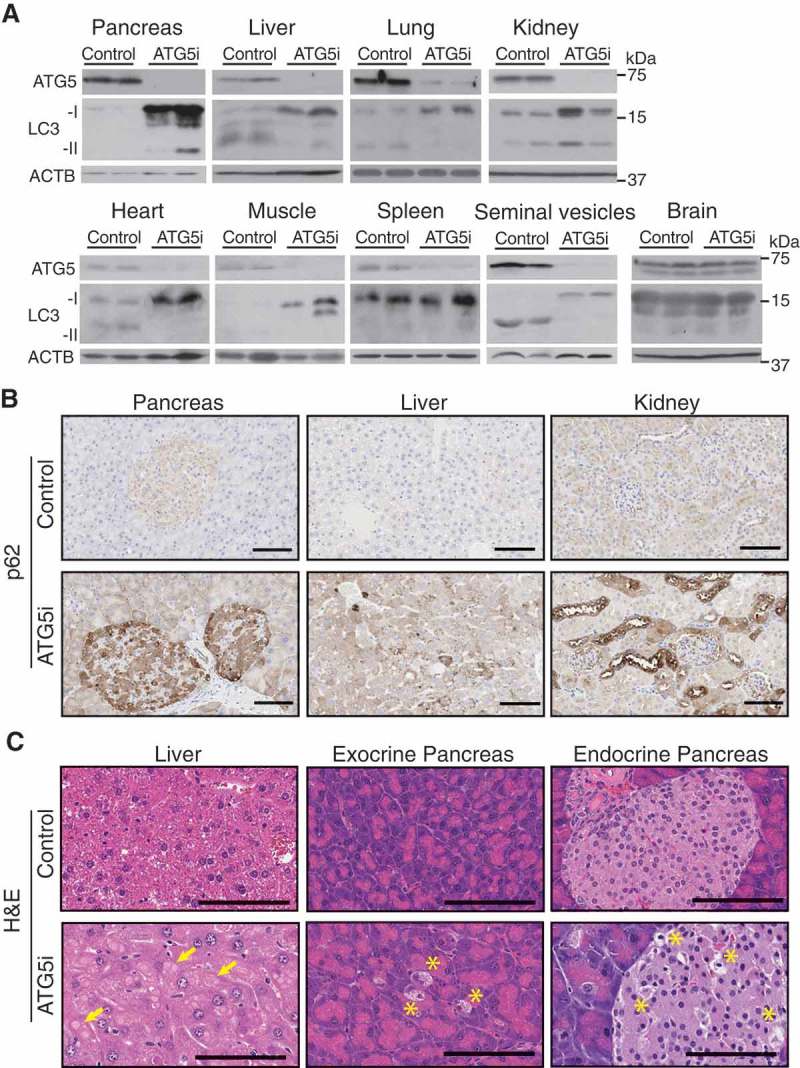
In vivo validation of autophagy inhibition upon *Atg5* downregulation. (a) ATG5 shows by western blot a downregulation across a range of tissue in adult mice treated with dox for 6 weeks, except the brain, which displays no alterations in ATG5 in whole tissue extracts. ACTB serves as a loading control in all tissues except for heart and muscle for which total actin was used instead. (b) ATG5i mice display an increase in SQSTM1 in the indicated tissues via IHC. (c) *Atg5* downregulation is associated with the development of large proteinaceous aggregates in the liver (yellow arrows). Additionally, cellular degeneration of the exocrine and endocrine pancreas is visible by H&E analysis (yellow asterisk). Scale bars: 100 μm in b and c.

We next validated *Atg5* knockdown in tissues from 6-week dox-treated ATG5i mice. Western blotting displayed a strong reduction in ATG5 levels as well as reduction in the conversion of soluble LC3-I to membrane bound LC3-II (Figure 2(a)). This was reflected in immunohistochemistry (IHC) analyses by the accumulation of SQSTM1, an autophagy receptor and substrate, forming aggregates to various degrees depending on tissue/cell type, with a heterogeneous and modest pattern in the spleen (Figure 2(b) and Figure S2). As reported previously, the increase of poly-ubiquitinated proteins was evident particularly in muscle and heart (Figure.S2B) [19]. In addition, pathological features of autophagy deficiency were also reproduced: e.g., in liver, hepatocytes were enlarged with intracellular proteinaceous aggregates, and in the pancreas, acinar and islet degeneration was noted in the dox-treated ATG5i mice (Figure 2(c)). These results indicate that the single copy integration of *shAtg5* in the genome is sufficient for robust downregulation of *Atg5* and, as a result, of autophagy activity *in vivo*.

One notable exception, however, was brain tissue, where we failed to detect any alterations in ATG5 and poly-ubiquitinated protein levels (Figure 2(a) and S2B). This is consistent with the reported inefficient expression of shRNA in the brain using this system [12]. Additionally, in contrast to whole-body somatic *atg7* KO mice, which develop lethal neurodegeneration [10], ATG5i mice displayed no evidence of overt neurological or motor phenotypes, and presented with normal limb clasping reflexes and brain histology when treated with dox for up to 8 months (Fig. S3). Despite this exception, ATG5i mice recapitulate many of the major phenotypes associated with autophagy deficiency at cellular, organ, and organismal levels.

**Figure 3.**
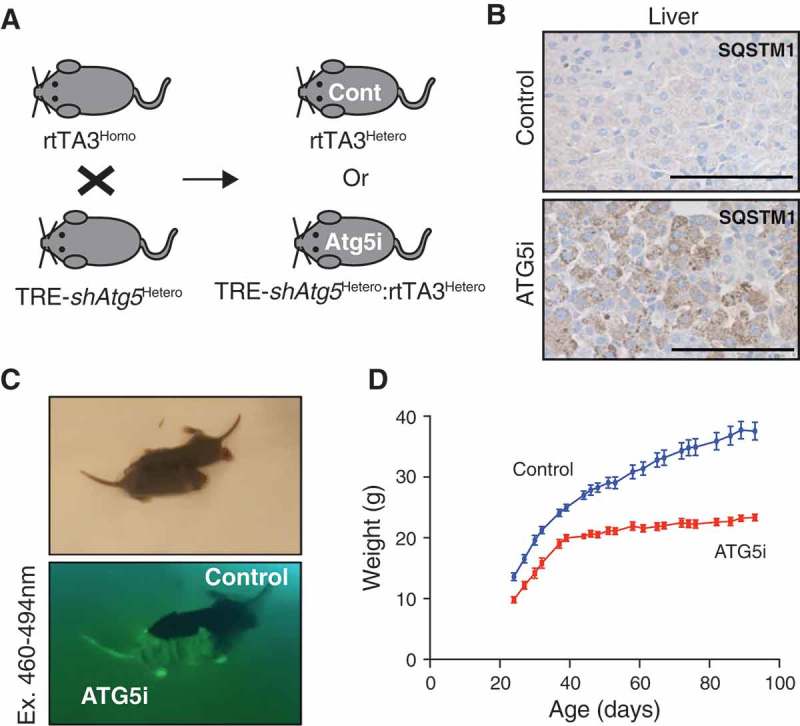
Perinatal survival of ATG5i neonates. (a) Breeding strategy for the generation of ATG5i neonates. A doxycycline-containing diet was fed to the parents who each have 1 component of the 2-component system, and thus are unable to induce *Atg5*-shRNA. Any embryos with both components, however, will induce the system. Cont, control. (b) IHC analysis of 14-day-old neonates highlights the presence of SQSTM1 aggregates in the livers of ATG5i mice in comparison to control. Scale bars: 100 μm. (c) ATG5i neonates are born at, and survive at, expected Mendelian ratios. They appear indistinguishable from their littermate controls except for their positivity for tGFP (bottom). (d) While initially indistinguishable, ATG5i mice do not show the same growth kinetics (n = 6 males in both conditions).

To test the resilience of ATG5i mice to survive the perinatal starvation period, *shAtg5* (homozygous) mice were crossed with *rtTA3* (heterozygous) mice and fed a dox-containing diet (Figure 3(a)). Only resultant offspring inheriting both components of the system are able to induce *shAtg5* expression. Consistent with embryonic *atg5* KO mice, ATG5i mice were born at close-to-expected Mendelian ratios (observed 40%; expected 50%) [8]. However, unlike *atg5* KO mice, all embryonic ATG5i mice were able to survive the neonatal starvation period. Similar results were recently described wherein restoration of *Atg5* expression, ectopically driven from a rat neuron-specific enolase promoter (*Eno2/NSE*), was sufficient to rescue neonatal lethality of conventional *atg5* KO mice, suggesting that the neonatal lethality of embryonic *atg5* KO mice is primarily due to neurological dysfunction including a suckling defect [8,18]. Although it is formally possible that this lack of neonatal death phenotype in the ATG5i model is due to hypomorphism of autophagy deficiency, IHC analyses showed strong accumulation of SQSTM1 aggregation in neonatal liver (Figure 3(b)). In addition, and congruent with previous publications, whereas ATG5i neonates appeared indistinguishable at birth to their littermate counterparts, their postnatal development of body size and weight was severely impaired (Figure 3(c,d)). These data reinforce the developmental role of autophagy in neuronal tissues, which is essential for survival during periods of neonatal metabolic stress [18].

**Figure 4.**
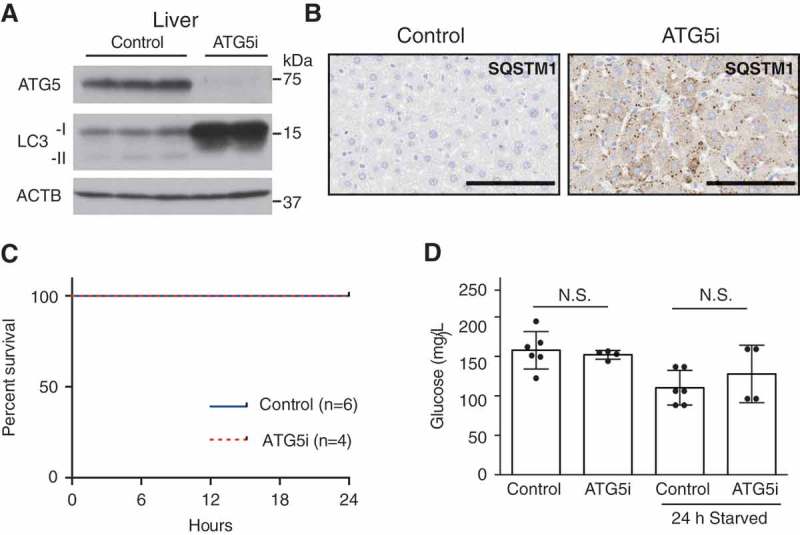
Adult ATG5i mice survive starvation-induced stress and maintain glucose homeostasis. (a) ATG5i mice administered dox for 2 weeks and having food withdrawn for 24 h prior to sacrifice display downregulation of ATG5 and a reduction of LC3-I to LC3-II conversion in the liver. (b) These same livers also show the formation of SQSTM1 aggregates as seen through immunohistochemical analysis. Scale bars: 100 μm. (c) These mice show no evidence of starvation-induced mortality when food is removed for 24 h (control, n = 6; ATG5i, n = 4). (d) Blood glucose levels before and after food withdrawal show no significant difference between control and experimental (free feeding P = 0.66; starved P = 0.37, Mann Whitney). N.S., not significant.

Autophagy has a critical role in maintaining energy homeostasis during periods of starvation-induced stress. Eighty percent of whole-body somatic *atg7* KO mice die with lethal hypoglycemia during a 24-h fasting period [10]. To test whether the ATG5i mice also recapitulate this phenotype, 8-week old ATG5i mice were treated with dox for a period of 2 weeks and then fasted for 24 h with free access to water, to replicate the same experimental design as previously reported [10]. At this time point, diminished expression of ATG5 in comparison to control mice was associated with SQSTM1 aggregation in the liver (Figure 4(a,b)). Unlike whole-body somatic *atg7* KO mice [10], ATG5i mice displayed no evidence of fasting-induced death (Figure 4(c)) and maintained blood glucose levels similar to that of control mice, despite continued suppression of ATG5 at least in the liver (Figure 4(d)). These results indicate that both embryonic and somatic ATG5i mice are highly robust under metabolic stress conditions, although it remains to be elucidated whether or not the unaltered autophagy activity in the brain of ATG5i mice is also responsible for rescuing starvation-induced death in the somatic model. Furthermore, these results reinforce the unique nature of the ATG5i model, which provides an opportunity for longer-term experiments involving autophagy-defective adults.

**Figure 5.**
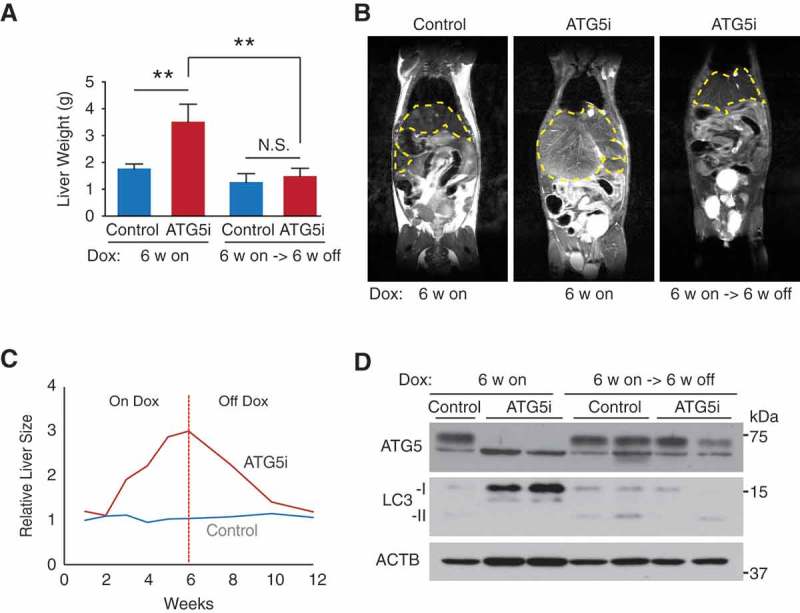
Restoration of ATG5 is associated with reversal of hepatomegaly. (a) Consistent with the results in Figure 1(f), adult mice treated with dox for 6 weeks develop hepatomegaly in comparison to control mice (P = 0.0035). However, the restoration of ATG5 levels in ATG5i mice is associated with a significant reduction in liver size (P = 0.007) to a weight similar to control mice on the same feeding regimen. (n = 8–10 mice per group; Kruskal-Wallis with Dunn’s post test, **P < 0.01, N.S., not significant) (b) Example images of an MRI scan from ATG5i mice at the 6-week on dox time point, as well as the 6-week on dox -> 6-week off dox time point displaying hepatomegaly and reversal to normal size, respectively. Yellow dotted lines encircle livers. (c) Time series analyses of liver size after dox addition, followed by dox withdrawal using MRI (n = 2 mice per condition, average value is shown; see Figure S5B for individual data). (d) Adult ATG5i mice fed on a dox-containing diet for 6 weeks display a downregulation of ATG5 and an increase in LC3-I by western blot analysis. When switched back to a normal diet for 6 weeks mice show a recovery in ATG5 levels and LC3-I, similar to control mice.

Next, we examined the effects of restoring autophagy in the ATG5i mice by taking advantage of the regulable nature of the system. As in Figure 2, 8-week old ATG5i and control mice were fed a dox-infused diet for 6-weeks to induce whole-body *Atg5* deficiency. At this point, mice were then switched to a standard diet (absent of dox) for further 6 weeks. Within this time window at least, the extent to which the *Atg5* knockdown-associated phenotypes recover upon *Atg5* restoration varied depending on the tissue type (Fig. S4 and S5). Strikingly, during necropsy, livers from these *Atg5*-restored mice were found to display no evidence of hepatomegaly (Figure 5(a)). The complete reversibility of hepatomegaly was confirmed through a time-course analysis using MRI imaging (Figure 5(b,c) and S5B). Re-expression of ATG5 was confirmed at the protein level by western blot analysis and was associated with a normalization of LC3 levels (Figure 5(d)), suggesting that autophagic flux had been re-established.

**Figure 6.**
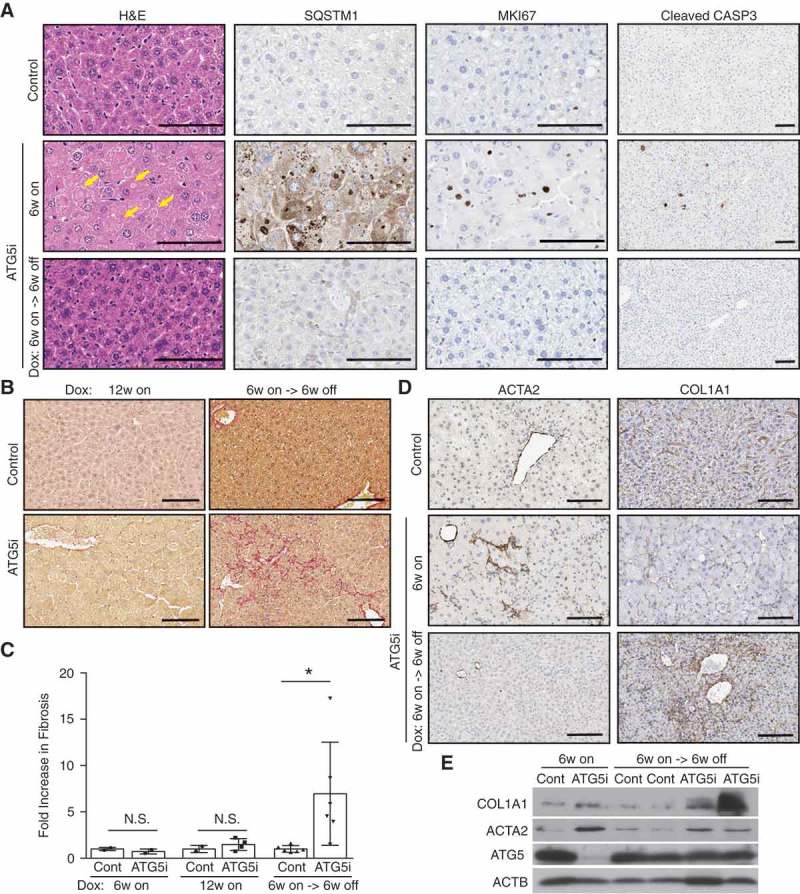
Restoration of ATG5 is associated with the induction of hepatic fibrosis. (a) Representative histology and IHC from the livers of control and ATG5i mice. Yellow arrows highlight large intracellular inclusions only found in ATG5i mice on dox. Scale bars: 100 μm. (b) Representative images of picrosirius red staining in sections of control and ATG5i mice in the indicated conditions. Scale bars: 100 μm. (c) Only ATG5i mice treated with dox for 6 weeks and off dox for 6 weeks showed the presence of fibrosis (P = 0.0468 Welch’s t-test, error bars represent s.d. around the means). **P < 0.01; N.S., not significant. Control (cont). (d) Immunohistochemical analysis of livers from each time point highlights that ACTA2/α-SMA-positive activated stellate cells are only present during the 6-week on dox time point, with staining positivity only present in the 6-week on dox->6-week off dox time point. (e) Whole tissue protein extracts display a similar trend with COL1A1 present only in the ATG5i mice at the 6-week on dox -> 6-week off dox time point.

Additionally, the histopathological alterations associated with autophagy deficiency, such as swollen hepatocytes and increased proliferation and apoptosis [5], were absent in the *Atg5* reconstituted livers (Figure 6(a) and Figure S6A and B). Evidence of liver damage and impaired liver function in ATG5i mice 6 weeks on dox, as measured by elevated serum GPT (glutamic pyruvic transaminase, soluble) and reduced serum ALB (albumin) levels, also reverted to control levels upon dox withdrawal (Fig. S6C and D).

Interestingly, we found that autophagy restoration in the liver was also associated with the induction of hepatic fibrosis as determined by picrosirius red (Figure 6(b,c)). This effect was not seen in ATG5i mice fed dox continuously for 6 or 12 weeks, suggesting that the increased fibrosis is not an outcome of autophagy deficiency *per se* but likely to be a secondary effect of autophagy restoration. This phenotype was also recapitulated in a short-term setting, where autophagy was restored after dox addition for 3 weeks (around which point hepatomegaly became evident as shown in Figure 5(c)), although the induction of fibrosis following 3 weeks off dox was modest, showing only a 2.5-fold increase in collagen staining (Fig. S7), in comparison to a 6.96-fold increase in the 6-week on/off regimen (Figure 6(c)). We next stained for ACTA2/α-SMA, a marker of activated hepatic stellate cells (HSCs), mediators of liver fibrosis, and found a substantial increase of ACTA2 after 6-weeks on dox in ATG5i mouse livers (Figure 6(d,e)). However, consistent with the picrosirius red staining (Figure 6(c)), COL1A1, a major component of liver fibrosis, did not show any increase in these same livers, but was instead upregulated only after autophagy restoration (Figure 6(b-d)). Note the major source of COL1A1 in the liver is activated HSCs; thus, our data suggest that whereas HSCs can be activated in the absence of autophagy, they are not fibrogenic. Interestingly, inhibition of autophagy, either genetically or pharmacologically, in HSCs can prevent COL1A1 expression and fibrosis during liver injury [20,21]. Thus, it is possible that in the ATG5i mice, systemic autophagy deficiency triggers hepatocyte damage, which activates HSCs, but the activated HSCs are not fully functional and the restoration of autophagy enables the primed HSCs to perform their functional roles, including the deposition of collagen. Thus, although pathological features of autophagy deficiency in the liver are largely reversible, transient autophagy inhibition may confer unforeseen adverse effects.

**Figure 7.**
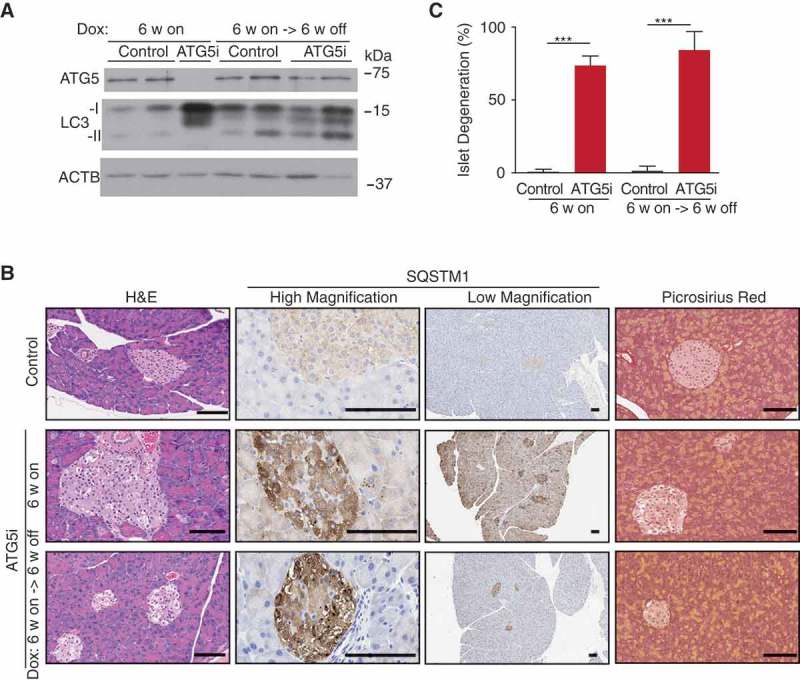
ATG5 restoration in the pancreas leads to partial phenotypic rescue. (a) Adult ATG5i fed on a dox-containing diet for 6 weeks display a downregulation of ATG5 and an increase in LC3-I by western blot analysis. When returned to a normal diet for 6 weeks mice show a recovery in ATG5 and LC3-I levels, similar to those seen in control mice. (b) Representative histology, IHC, and picrosirius red staining (marker of fibrosis) from the pancreas of control and ATG5i mice. Whereas SQSTM1 levels are increased in both the islets and acinar of ATG5i mice on dox, only the acinar display a reversal once dox is removed. Islets do not show a reversal of the degeneration phenotype as viewed by H&E. Scale bars: 100 μm. (c) Dox-treated adult mice display evidence of islet degeneration that is not reversed upon dox withdrawal. Mann-Whitney test, ***P < 0.001.

In the pancreas, the removal of dox was also associated with the re-expression of ATG5 and near complete normalization of LC3 levels as evidenced by whole-tissue western blot analysis (Figure 7(a)). However, whereas SQSTM1 levels were elevated in both acinar and islet compartments during dox administration in ATG5i mice, only the acinar cells of the pancreas displayed a normalization of SQSTM1 levels upon dox removal (Figure 7(b)). Consistently, whereas the acinar portion of the pancreas histologically recovered, the islets still appeared degenerative with areas of vacuolization apparent (Figure 7(b,c)). Thus, the data suggest that, similar to the liver, autophagy deficiency-associated phenotypes of the pancreatic acinar are also reversible. However, in contrast to the liver, the pancreas displayed no evidence of fibrosis after *Atg5* restoration (Figure 7(b)). The reason for the observed irreversibility of the islet phenotype is unclear. Of note, the acinar was found to display evidence of increased proliferation that was not seen in the islets and may reflect the natural abilities of these cellular populations to recover after stress (Fig. S8).

## Discussion

Here we report the temporally-regulable mouse model of autophagy, enabling both downregulation, and subsequent re-expression of endogenous *Atg5 in vivo*. Although this model can be both systemic and tissue-specific, in this study, we focused on the systemic model to evaluate the overall effects of the system. As previously described with this system [12], ATG5i mice exhibited no apparent *Atg5* knockdown in brain. Interestingly, although further detailed analyses are required to determine the knockdown efficiency in entire tissues/cell types, the lack of *Atg5* knockdown in brain has created a unique situation analogous to the recently developed *Atg5*^−/-^;*Eno2/NSE-Atg5* mice. In these mice, *Atg5* is ectopically expressed under a neuron-specific promoter in the conventional *atg5* KO background [18]. In contrast to *Atg5*^−/-^;*Eno2/NSE-Atg5* mice, which is an embryonic system, in ATG5i mice the shRNA can be induced either embryonically or somatically, the latter is particularly useful to separate developmental phenotypes from the role of autophagy in tissue homeostasis. Thus, together with its dynamic nature, the ATG5i mouse model offers a unique and complementary resource for autophagy studies. However, it is important to note any possibility of off-target effects of the RNAi in this system. Whereas our model exploits a recently developed inducible shRNA system, wherein the shRNA is expressed in a miR-E cassette to allow physiological processing and reduced off-target effects [22], currently we only use one targeting sequence. As such it will be important to develop further RNAi models targeting alternative sequences of *Atg5*, or other key autophagy genes, for further validation of newly described phenotypes.

We report that ATG5i mice appeared refractory to metabolic stress unlike conventional and conditional autophagy knockout mice. Analogous to the *Atg5*^−/-^;Eno2/*NSE-Atg5* model, dox-treated ATG5i neonates (where dox was administered throughout embryogenesis) did not display the characteristic rapid perinatal lethality. Moreover, 24-h food withdrawal in adult ATG5i mice was not associated with a lethal hypoglycemic response, in contrast to somatic *atg7* KO mice, which develop extensive brain damage. Although, in our ATG5i model, it is not possible to study direct effects of dynamic autophagy modulation in brain, data from this model raise an interesting question as to whether basal autophagy in the adult brain plays a critical role in systemic metabolic homeostasis under starvation conditions.

We leveraged the dynamic nature of our system and show tissue- or cell type-specific differences in the reversibility of any alterations associated with autophagy deficiency, at least during the time range tested. Further studies will be required to determine the exact source of this heterogeneity and whether longer restoration times are required for some tissues. Nevertheless, our model showed near complete reversibility in the liver and pancreatic acinar. Importantly, in the liver, despite the full reversibility of pathologies derived from *Atg5* knockdown, autophagy restoration enhanced fibrosis. This does not appear to affect liver function (Fig S6C), but may alter the long-term fate of the tissue microenvironment. It is also possible that the length of the initial autophagy deficiency affects the liver function after autophagy restoration. Hepatocyte cell death due to autophagy inhibition has been previously reported to lead to the activation of HSCs and drive fibrosis [23]. It was also shown that *Atg5*-deficient macrophages facilitate chemically-induced liver fibrosis through stimulating myofibroblasts (likely to be activated HSCs [24]) to express fibrogenic genes [25]. In our ATG5i mice, however, despite hepatocyte cell death, HSC activation and *Atg5* knockdown in immune cells/macrophages (Fig. S9), the livers of ATG5i mice on dox failed to exhibit collagen deposition and fibrosis, which only developed after autophagy restoration (Figure 6), reinforcing the critical role of autophagy within activated HSCs for the fibrogenic activity of these cells [20,21].

In the clinical setting, autophagy-modulating therapies have garnered interest for life- and health-span modulation, as well as in the field of oncology [2,26,27]. Particularly for the latter, inhibition of autophagy (considered as a cytoprotective program) has generally been suggested for use in conjunction with standard chemotherapy. As such, the temporal modulation of autophagy is considered a rational goal to achieve clinical benefit. However, regimens to date that modulate autophagic flux do not act specifically on the autophagy machinery. Instead they often target other components of the cellular system to alter autophagy, and as such distinguishing autophagy-specific effects is often difficult. Our data suggest that the systemic ATG5i mice may be utilised to model specific anti-autophagy therapies. Additionally, there is very little understanding of the potential adverse effects of switching systemically from an autophagy-low state to an autophagy-high or restored state, which, as highlighted here, may be associated with further complications. As shorter regimens of autophagy inhibition appear to result in reduced fibrosis in this system, we speculate that the timing of dosing, as well as the degree of autophagy inhibition, may be a critical determinant in the generation of pathological effects.

## Methods

### Antibodies

#### For western

Anti-ATG5 (Abcam, ab108327; 1:1000), anti-LC3 (Nanotools, Clone 5F10; 1:1000), anti-ACTB (Sigma, A5441; 1:10,000), anti-ACTIN (Santa Cruz Biotechnology, I-19; 1:5000 [no longer commercially available]), anti-ACTA2/α-SMA (Abcam, ab5694; 1:1000), anti-COL1A1 (Abcam, ab34710; 1:2000), anti-poly UBIQUITIN (Enzo, Clone FK1; 1:5000), anti-turboGFP (Pierce, PA5-22,688; 1:2000), anti-rabbit HRP and anti-mouse HRP (GE Healthcare, NA934V and NA931V; 1:5000)

#### For immunohistochemistry (IHC)

Anti-SQSTM1 (Enzo, BML-PW9860; 1:750), anti-MKI67 (Abcam, ab16667: 1:1000), anti-cleaved CASP3/caspase 3 (Cell Signalling Technology, 9664; 1:200), anti-ACTA2/α-SMA (Abcam, ab5694: 1:500), anti-COL1A1 (Abcam, ab34710; 1:1000).

### Western blot analysis

Western blot analysis was performed as previously described [28]. Cells and tissues were lysed in Laemmli buffer; for tissues samples were homogenised with the Precellys 24 tissue homogeniser (Precellys) in Laemmeli buffer. Samples were run on 12.5% or 15% gels and transferred to PVDF membranes (Immobilon: Millipore, IPVH00010). The membrane was blocked for 1 h at room temperature with 5% milk solution in TBS-Tween (Tris-buffered saline [50 mM Tris, 150 mM NaCl, pH 8.0] containing 0.1% Tween 20 [Fisher BP337-500]) before incubating with primary antibody at 4°C overnight. Subsequently, an appropriate HRP-conjugated secondary antibody was added followed by incubation at room temperature for 1 h. Western blots were visualised with chemiluminsence reagents (Sigma, RPN2106).

### IHC

Formalin-fixed paraffin-embedded samples were de-waxed and rehydrated before antigen unmasking with citrate buffer (10 mM sodium citrate, 0.05% Tween 20, pH 6) in a pressure cooker for 5 min at 120°C. Remaining steps were according to the Dako Envision+ Rabbit kit (K4010) instructions.

### Picrosirius red staining and quantification

Briefly FFPE slides were de-waxed and rehydrated before being stained with Weigers Haematoxylin (8 min; CellPath RBA-4201-00A), washed in running water (10 min), immersed in picrosirius red solution (0.1% [w:v] Direct Red 80 [Sigma 365,548] in a saturated picric acid solution [Sigma P6744]) for 1 h and washed in acidified water (0.5% acetic acid) for 2 changes. Slides were then dehydrated and cover slipped. Once stained, images were taken at random using a Nikon T-2000 inverted microscope and DSFi-1 camera. The specimen was illuminated with circularly polarised light by setting the de Senarmont compensator of the Nikon microscope with the polariser at 45 degrees to the fast/slow axes of the quarter-wave plate. A circular polariser was placed in the light path from the objective lens, producing a dark field, as its circular polarization was opposite to that of the illuminating beam. In this condition, the birefringent specimen is essentially placed between crossed circular polarisers and remains bright at all azimuthal positions. If an area was found to overlap with a large vessel it was discounted and images not taken for analysis, in all conditions at least 10 images were used for analysis. Subsequently, images were analyzed in Fiji by generating a threshold in a control sample and using this across all samples. The remaining area count provided ‘positive fibrotic area’. Each sample was then normalised to the mean value of all the controls.

### Blood glucose homeostasis during starvation

Blood glucose measurements were taken prior to, and after 24 h of, food withdrawal using the ACCU-CHEK Aviva blood glucose monitor. As doxycycline is provided in the diet of mice (Test Diets, 5A5X), mice also received doxycycline intraperitoneally (20 ml/kg of a 4 mg/ml solution, Sigma, D9891) to ensure continued expression of *shAtg5*.

### MKI67 counting

For livers, automated counting of MKI67 across the entire liver section was performed using ImageScope^TM^ (Leica Biosystems) customised for the liver and reported as a percentage of nuclei that stained positively. For the pancreas, acinar and islets were counted separately using HALO^TM^ software (Indicalab) and reported as a percentage of nuclei that stained positively.

### Cleaved CASP3 analysis

For livers, automated counting of stain positivity across the entire liver section was performed using ImageScope^TM^ (Leica Biosystems) customised for the liver and reported as a percentage of pixels in the image that stained positively, known as a positive pixel count.

### Liver function tests

Sera isolated from mice were analyzed by the Core Biochemical Assay Laboratory (CBAL), Cambridge, UK. Samples were run on an automated Siemens Dimension RxL analyzer for GPT (Siemens Healthcare) and ALB (Siemens Healthcare).

### Islet degeneration

Each pancreas had all islets available in the histological section imaged and counted for the presence or absence of islet degeneration. The number of degenerative islets was calculated as a percentage of the total number of islets visible on 1 section from a single mouse. This was calculated for control and ATG5i mice and a mean and standard deviation was calculated for each group and displayed graphically.

### MRI

MRI scans were acquired on a 9.4 T Agilent MRI scanner running VnmrJ 3.1A and equipped with gradients of maximum strength 40 Gauss/cm and inner diameter 120 mm. A quadrature millipede coil of inner diameter 40 mm was used for all imaging studies. Coronal fast spin-echo images were acquired with and without fat suppression employing a chemical-shift-selective sinc-profile radio frequency pulse. Slice thickness was 1 mm, field of view 100 mm x 50 mm, 512 × 256 points providing in-plane resolution of 200 µm. Up to 18 slices were acquired to cover the full body of the mouse. The effective echo time was 44 ms, echo train length 8, and 4 averages were acquired to improve signal-to-noise ratio in the liver, which is hypointense with these parameters. The nominal repetition time was 3 sec, but in practice this was determined by the respiratory gating employed to minimise motion artifacts in the upper abdomen. This sequence gives good contrast between different soft tissues. Organ volumes were measured by drawing regions of interest on the fat-suppressed images. Fat volume was assessed by subtracting the unsuppressed images from the suppressed images and thresholding the resulting image.

### Generation and maintenance of Atg5i mice

A panel of shRNAs in a microRNA backbone (miR30 design) [12] targeting *Atg5* was obtained from Mirimus Inc. through a sensor-based screening system [14] in a pLMP [12] backbone and was used to generate retroviral supernatant, except for *shAtg5* #4 (pMSCV), which was previously used (targeting both human *ATG5* and mouse *Atg5*) [28]. We tested knockdown efficiency of those shRNAs in NIH3T3 cells, and an shRNA showing the strongest knockdown even with the highest dilution (1% v:v) viral supernatant was taken forward for LSL-ATG5i mouse generation by Mirimus Inc. Briefly, the shRNA (*Atg5*_*1065;* Guide sequence: TATGAAGAAAGTTATCTGGGTA) in a miR-E design (an improved design variant of miR30) [22] was inserted downstream of the *Col1a1* locus via recombinase-mediated cassette exchange which enables efficient targeting of a transgene to a specific genomic site 500 base pairs downstream of the 3ʹ UTR in D34 ES cells expressing *CAG-rtTA3* knocked into the *Rosa26* locus (Fig. S1) [29,30]. Mice were maintained on a mixed C57Bl/6 X 129 background and littermate controls were used in all experiments. All experimental mice were maintained as heterozygous for both the *Atg5_1065* and *CAG-rtTA3* alleles, whereas control littermates were lacking one of the alleles. Mice were maintained in a specific pathogen-free environment under a 12-h light/dark cycle, having free access to food and water unless otherwise stated. Mice were fed either a laboratory diet (PicoLab Mouse Diet 20, 5R58) or the same diet containing doxycycline at 200 ppm (PicoLab Mouse Diet, 5A5X). All experiments were performed in accordance with national and institutional guidelines, and the study was approved by the ethics review committee of the University of Cambridge. Imaging of neonates for tGFP expression was conducted using an excitation lamp (460–494 nm) and emission filter (500–515 nm) (BLS, FHS/LS-1B) optimised for fluorescent proteins in the green wavelength.

### RNAi target sequences

The following shRNAs in the pLMP vector were used for their in vitro validation. Guide sequences (antisense) are bold in a miR30 context presented as XhoI-EcoRI fragments.

*shAtg5* #1 (Atg5_1065):

CTCGAGAAGGTATATTGCTGTTGACAGTGAGCGCACCCAGATAACTTTCTTCATATAGTGAAGCCACAGATGTA**TATGAAGAAAGTTATCTGGGTA**TGCCTACTGCCTCGGAATTC

*shAtg5* #2 (Atg5_1068):

CTCGAGAAGGTATATTGCTGTTGACAGTGAGCGACAGATAACTTTCTTCATATTATAGTGAAGCCACAGATGTA**TAATATGAAGAAAGTTATCTGG**TGCCTACTGCCTCGGAATTC

*shAtg5* #3 (Atg5_1654):

CTCGAGAAGGTATATTGCTGTTGACAGTGAGCGCCAGTGAGAGATTTTTAAATAATAGTGAAGCCACAGATGTA**TTATTTAAAAATCTCTCACTGT**TGCCTACTGCCTCGGAATTC

*shAtg5* #4:

CTCGAGAAGGTATATTGCTGTTGACAGTGAGCGCCTTTGATAATGAACAGTGAGATAGTGAAGCCACAGATGTA**TCTCACTGTTCATTATCAAAGT**TGCCTACTGCCTCGGAATTC

*shRenilla* (Ren_713):

GAAGGCTCGAGAAGGTATATTGCTGTTGACAGTGAGCGCAGGAATTATAATGCTTATCTATAGTGAAGCCACAGATGTA**TAGATAAGCATTATAATTCCTA**TGCCTACTGCCTCGGAATTC

### Liver immune cell isolation

Dissected livers were homogenised (Miltenyi Liver Dissociation Kit, 130–105-807) and passed through a 70-μm filter. After centrifugation, red blood cells were lysed with RBC Lysis Buffer (eBioscience, 00–4300-54) for 10 min. After centrifugation, samples were washed twice in PEB buffer (phosphate-buffered saline [137 mM NaCl, 2.7 mM KCl, 10 mM Na_2_HPO_4_, 1.8 mM KH_2_PO_4_, pH 7.0], 5 µM EDTA, 0.5% BSA [Sigma, A7030]). Immune cells were enriched using an OptiPrep gradient (Sigma, D1556) and the ADGRE1/F4/80^+^ population isolated by incubating the immune cell population with ADGRE1/F4/80 MicroBeads (Miltenyi, 130–110-443) and passing the mixture sequentially through 2 MACs columns (Miltenyi, 130–042-201) sequentially. Purified macrophages were stained with Fixable Viability Dye eFluor^TM^ 780 (Thermo Fisher Scientific, 65–0865-14) to distinguish live cells from dead cells. Subsequently, cells were blocked with TruStain fcX™ (anti-mouse FCGR3/CD16-FCGR2B/CD32, clone 93; Biolegend, 101,320) antibodies and then stained with BUV395-conjugated antibody against PTPRC/CD45 (clone 30-F11; BD Biosciences, 564,279) and BV421-conjugated antibody against ADGRE1/F4/80 (clone BM8; Biolegend, 123,131). Stained cells were analyzed using FACS LSR II (BD) and acquired results were analyzed using FlowJo software (v10.4, FlowJo, LLC).

### RNA analysis

RNA was isolated using the Qiagen RNEasy Micro Kit (74,034), and cDNA generated using Superscript™ III Reverse Transcriptase (Invitrogen, 18,080–044) and random hexamers (48,190–011). Primer sequences were as follows:

***Atg5***: *Forward 5ʹ-GCCGAACCCTTTGCTCAATG-3ʹ*

Reverse 5ʹ-TGGTCACCTTAGGAAATACCCAC-3ʹ

***Actb****: Forward 5ʹ-CAAGAGAGGTATCCTGACCCTGAAG-3ʹ*

Reverse 5ʹ-CATTGTAGAAGGTGTGGTGCCAG-3ʹ
