# Supplementary material for: A novel Atg5-shRNA mouse model enables temporal control of Autophagy in vivo
Source: Autophagy. 2018 Jul 12;14(7):1256–66. doi: 10.1080/15548627.2018.1458172 (PMC6103714; doi:10.1080/15548627.2018.1458172)
Supplement: Supplemental Material [file kaup-14-07-1458172-s001.zip › Supplementary information/2017AUTO0313R2-s01.docx]

Dear Dan,

Please find attached our revised manuscript entitled “A Novel *Atg5*-shRNA Mouse Model Enables Temporal Control of Autophagy *in vivo*”

Thank you again for your careful editing and we apologize that we had left many typos in the previous version. We have now incorporated all the suggestions/corrections with some additional minor corrections (reference style etc.). Please note, in Fig. S1C, we have double checked with the original literature and the company Mirimus scientist about the diagram, and it turns out that the βcHS4 insulator was not used in this mouse ES cells. This insulator element has now been removed from the diagram. Thank you for bringing our attention to this.

Please let me know if you have any queries about the manuscript.

Sincerely,

Masashi
