# Supplementary material for: A novel Atg5-shRNA mouse model enables temporal control of Autophagy in vivo
Source: Autophagy. 2018 Jul 12;14(7):1256–66. doi: 10.1080/15548627.2018.1458172 (PMC6103714; doi:10.1080/15548627.2018.1458172)
Supplement: Supplemental Material [file kaup-14-07-1458172-s001.zip › Supplementary information/Suppl.pdf]

Cassidy_Figure S1

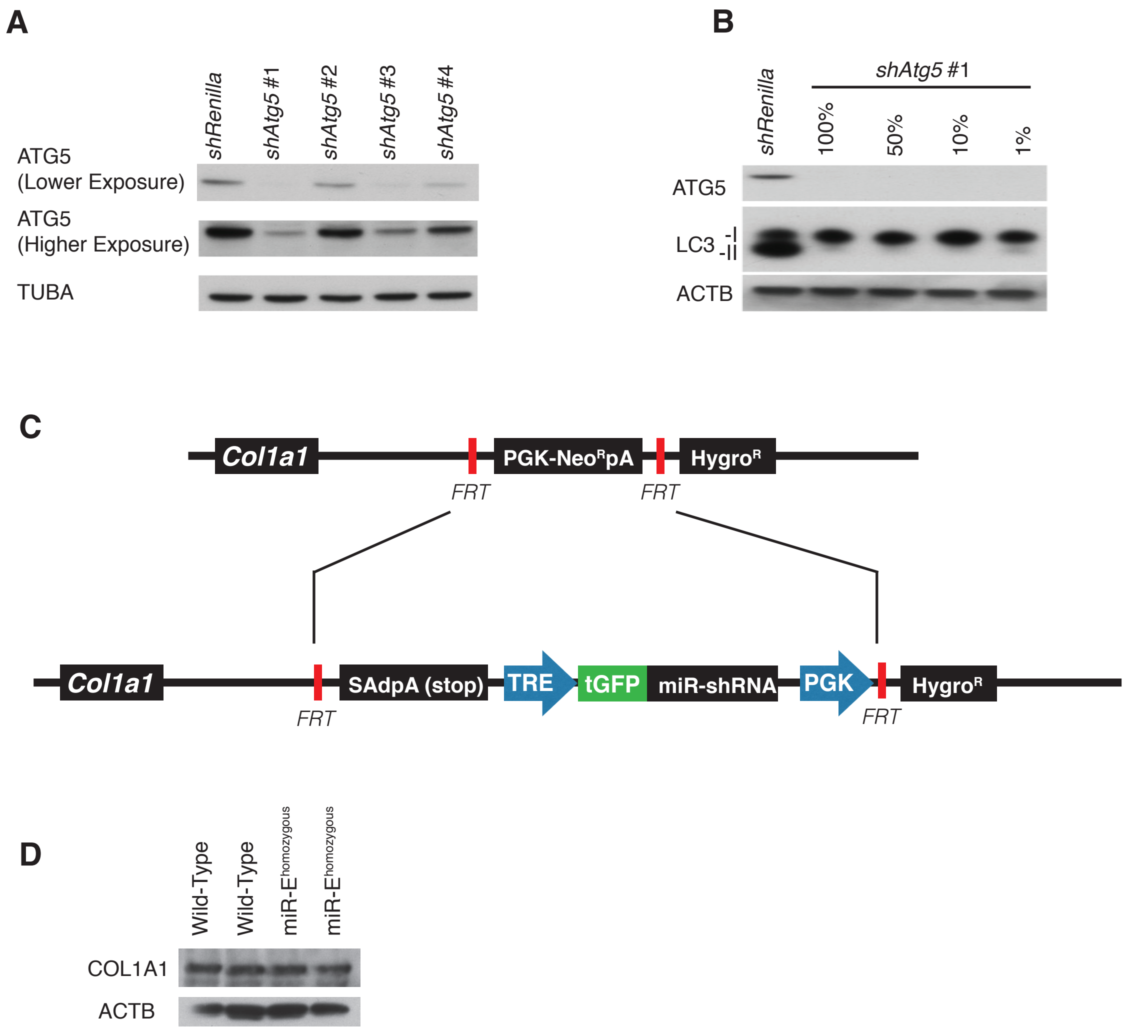

Figure S1. In vitro screening for potent shRNA targeting *Atg5.* (**A**) A panel of shRNAs was obtained from Mirimus Inc. in a pLPE backbone. Retrovirus for each shRNA were generated and knockdown efficiency tested in NIH3T3 cells. *shAtg5#1* (Atg5_1065) provided the greatest knockdown efficiency by western blot analysis during infection. (**B**) This occured at the lowest dilutions of retrovirus tested (1%), with a near complete abrogation of LC3-II conversion. Dilutions are shown as percent volume of the retroviral soup directly derived from packaging cell culture and diluted into the medium of NIH3T3 cells. (**C**) Schematic illustrates the relationship between the *Col1a1* gene and the shRNA cassette. Targetting of the shRNA was achieved using recominase-mediated cassette exchange at a specific site 500 base pairs downstream of the *Col1A1* 3'UTR in D34 ES Cells [29,30]. FRT: flippase recognition target. TRE: tet-responsive element. SAdpA (stop), splicing acceptor donor polyA sequence. HygroR: hygromycin resistance cassette. (**D**) Homozygous targeting of the shRNA does not alter COL1A1 expression in MEFs in comparison to littermate wild-type controls.

Cassidy_Figure S2

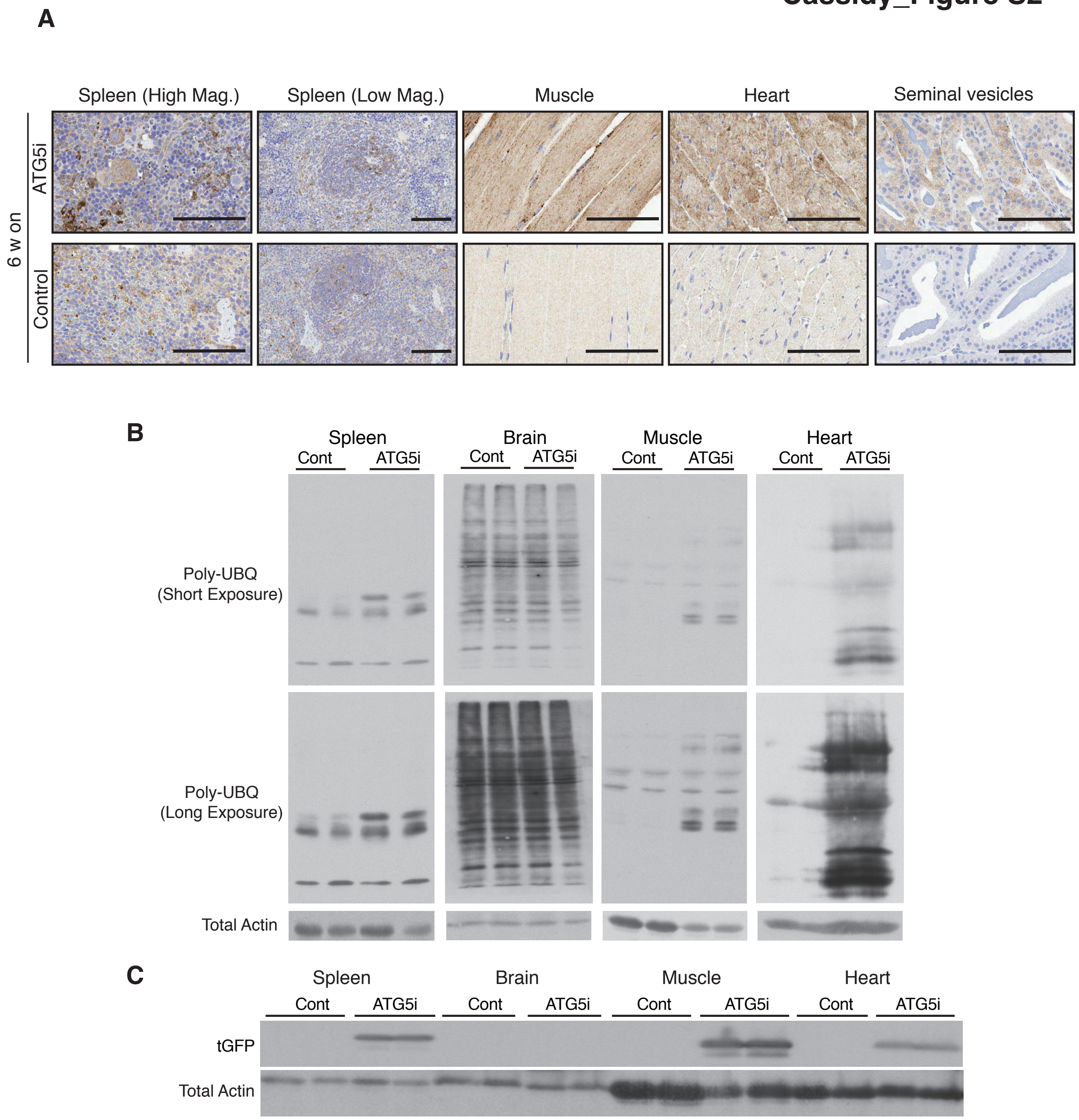

Figure S2. Extended characterization of ATG5i mice. (**A**) An increase in the staining for the autophagy adaptor protein SQSTM1 can be seen in muscle, heart and seminal vesicle tissue. Spleen tissue provides a heterogeneous staining even in the control (cont) tissue with an increase in intensity in the ATG5i mice. (**B**) Western blotting for poly-ubiquitin (UBQ) highlights an increase, particularly in heart and muscle. Brain tissue shows no alterations. (**C**) A single gel containing all four tissues (5µg loaded per lane) highlights the differences in the expression of tGFP, a marker of system activation. As expected brain tissue displays a much reduced expression due to the bioavailabilty of dox.

Cassidy_Figure S3

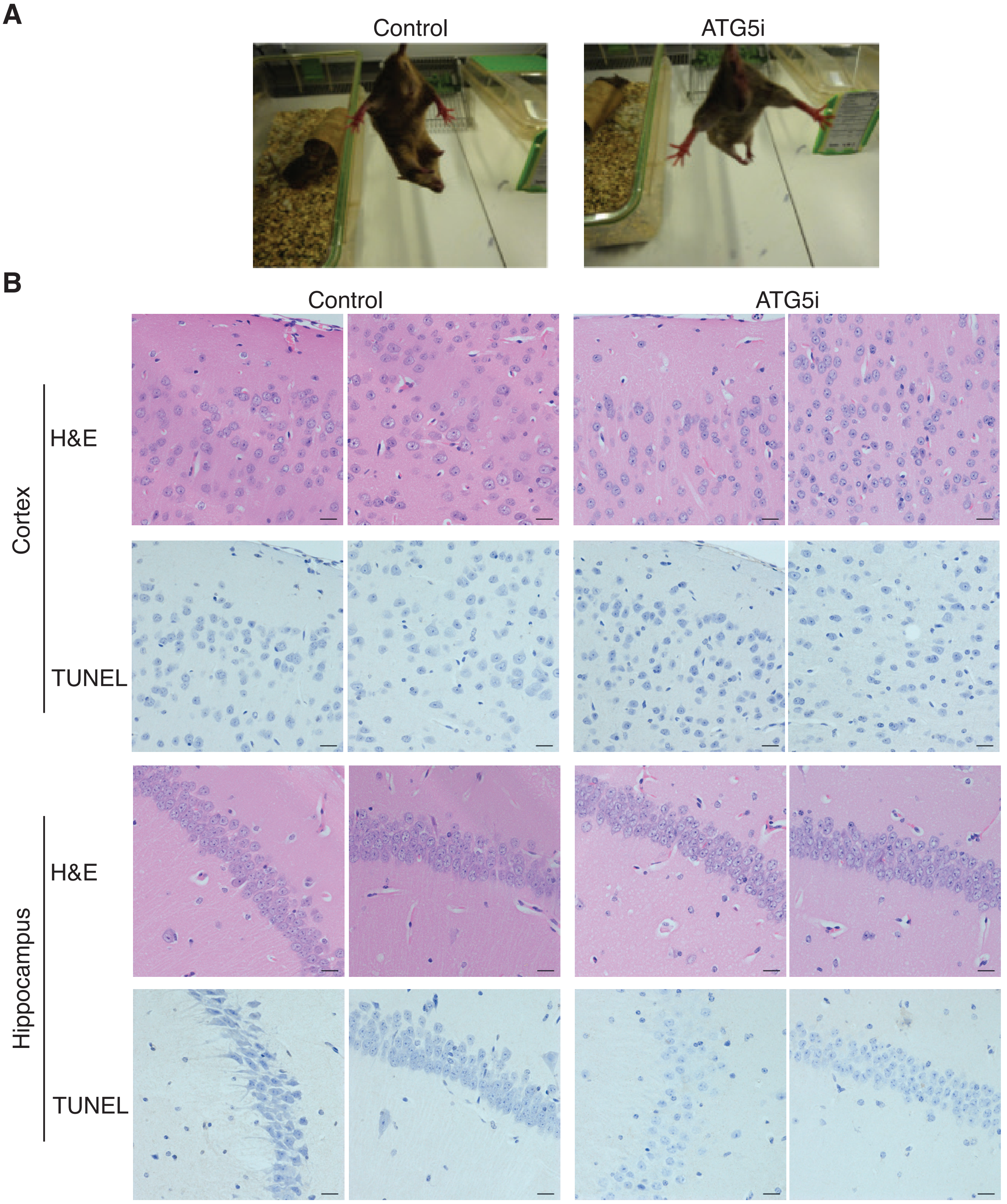

Figure S3. ATG5i mice display no evidence of neurotoxicity. (**A**) Adult mice fed on dox-based diet for 6-weeks display normal limb clasping reflexes in both control and ATG5i cohorts. Note the limbs are extended in both control and ATG5i mice, unlike limb clasping towards the body as seen in some models of neurodegeneration (**B**) Age-matched brain histology from mice treated with dox for 5-6 months (n=3 ATG5i and control mice). Sagittal sections (10 µm) were assessed blinded across various brain regions with particular emphasis on the cortex and hippocampus. Representative images are given for regions spanning cortical layers and hippocampal pyramidal CA2/3 region layers. Hematoxylin and eosin (H&E) staining showed no consistent differences between groups with a staining pattern reflective of aged mice, such as observable vacuolization. No above baseline TUNEL-positive staining was observed between groups. Scale bars: 20 µm.

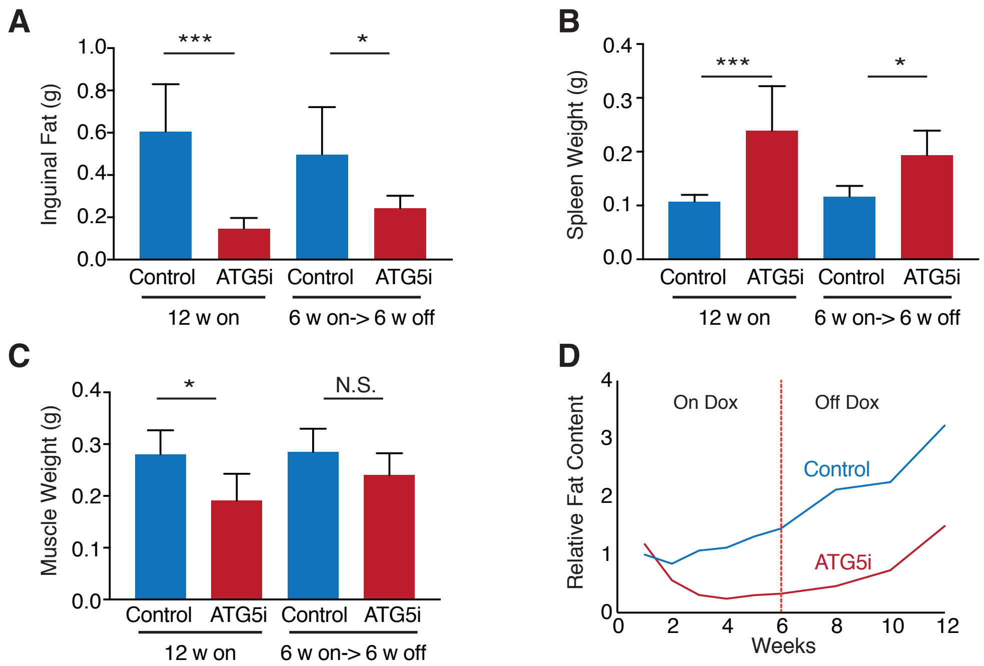

Figure S4. Reversibility of phenotype induced by ATG5 knockdown is tissue-type dependent. (**A**) Inguinal fat weights are significantly reduced in the ATG5i cohorts during dox administration and do not display a recovery in weight during the 6-week on dox -> 6-week off dox time point. (**B**) Similarly splenic weights are significantly different while on dox, and also do not appear to significantly recover at the 6-week on dox -> 6-week off dox time point. (**C**) Muscle weight was significantly different between control and ATG5i mice while on dox, however this significance was lost during the 6-week on dox -> 6-week off dox time point. Mann-Whitney test *P<0.05, **P<0.01, ***P<0.001; N.S, not significant. (**D**) Time series analyses of total body fat content after dox addition, followed by dox withdrawal using MRI (*n*=2 mice per condition, average value is shown; see Figure S5A for individual data).

Cassidy_Figure S5

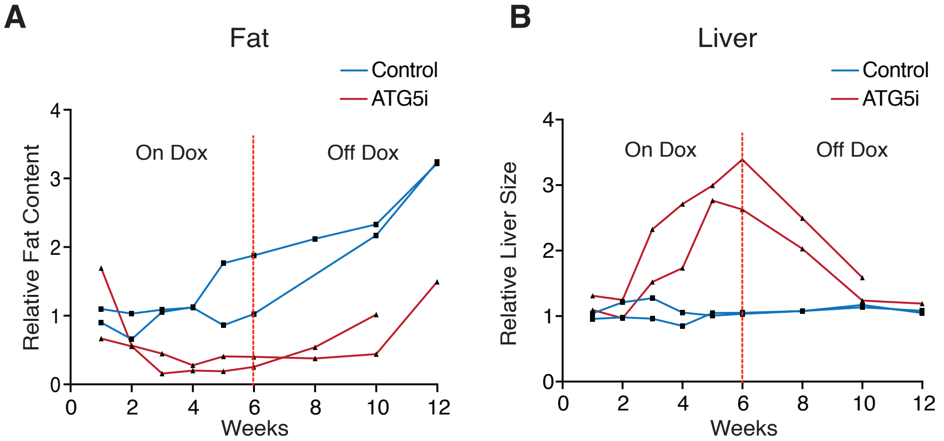

Figure S5. Dynamics of adipose tissue and liver during doxycycline administration. Individual data points from MRI studies of fat (Fig. S4D) and liver (Fig. 5C) in adult ATG5i fed on a dox-containing diet for 6-weeks, before being moved to a dox-free diet for 6-weeks. (**A**) Relative fat content and (**B**) Relative liver volume (n=2 mice per condition).

Cassidy_Figure S6

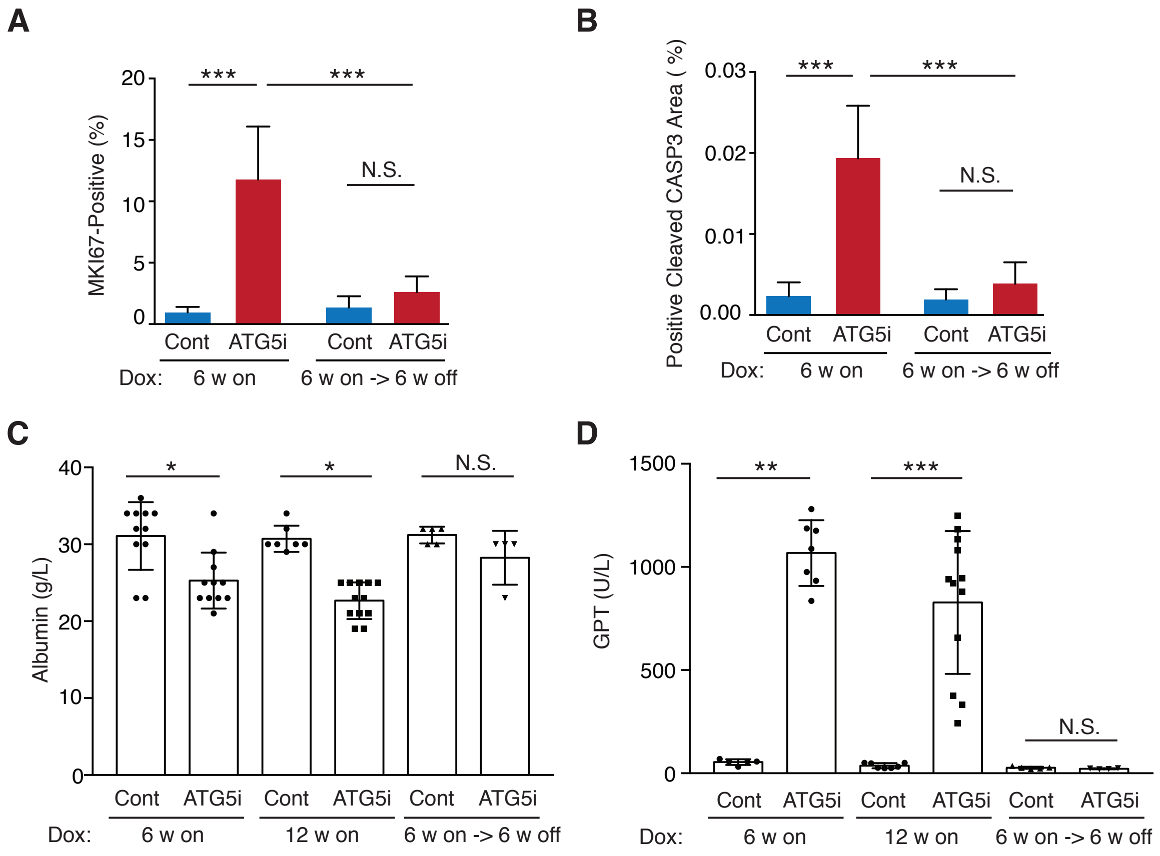

Figure S6. Liver functionality reflects ATG5 levels in vivo. (**A**) Proliferation, as measured through the presence of MKI67-positive cells in IHC, was increased after 6-weeks of dox in the ATG5i group in comparison to controls (cont) (P<0.001) and displayed a significant decrease upon *Atg5* restoration (P<0.001) to levels comparable to control mice. One-way ANOVA, *n*= 5-6 mice per condition. (**B**) Apoptosis, as measured by cleaved-CASP3 in IHC, is higher in ATG5i mouse livers during dox administration compared to control mice (P<0.001). Restoration of *Atg5* is associated with a reduction in cleaved-CASP3 positivity (P<0.001) and a return to basal levels. One-way ANOVA, n= 3-7 mice per condition. All error bars represent s.d. around the means. ***P<0.001; N.S, not significant. (**C**) Serum (ALB) albumin levels in ATG5i mice are suppressed in the presence of dox for 6 and 12 weeks in comparison to controls, P= 0.0460 and P=0.0116 respectively. Serum ALB levels recover to normal levels once dox is removed from the diet and *Atg5* levels are restored. Kruskal-Wallis Test. n= 4-12 mice per condition with individual values displayed. (**D**) Serum GPT levels are significantly increased during a 6-week (P<0.0025, Mann-Whitney) or 12-week period on dox in Atg5i mice (P<0.0001, Mann-Whitney), which was not seen in the 6 weeks on- 6 weeks off dox cohort. *P<0.05, **P<0.01, ***P<0.001; N.S, not significant.

Dynamics of adipose tissue and liver during doxycycline administration.

Cassidy_Figure S7

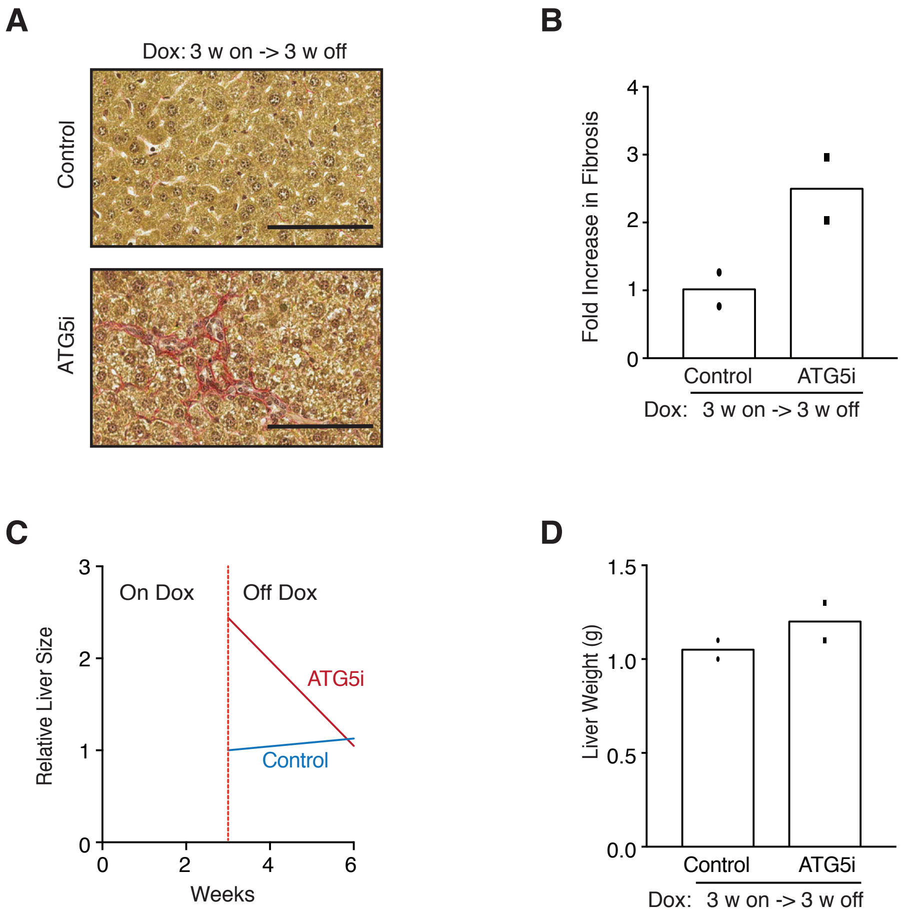

Figure S7. Short-term inhibition and restoration of ATG5 is also associated with liver fibrosis. (**A**) ATG5i mice treated with dox for 3 weeks and subsequently moved to a dox-free diet for 3 weeks show a similar fibrosis phenotype to the 6-week on dox->6-week off dox time point. (**B**) Quantification of picrosirius red staining at the end of this time course (**C-D**) ATG5i mice displayed a larger liver after 3 weeks of dox, which reduced in size to normal levels after dox had been removed as seen by MRI (**C**) and tissue weight upon death (**D**). Values are means of 2 mice.

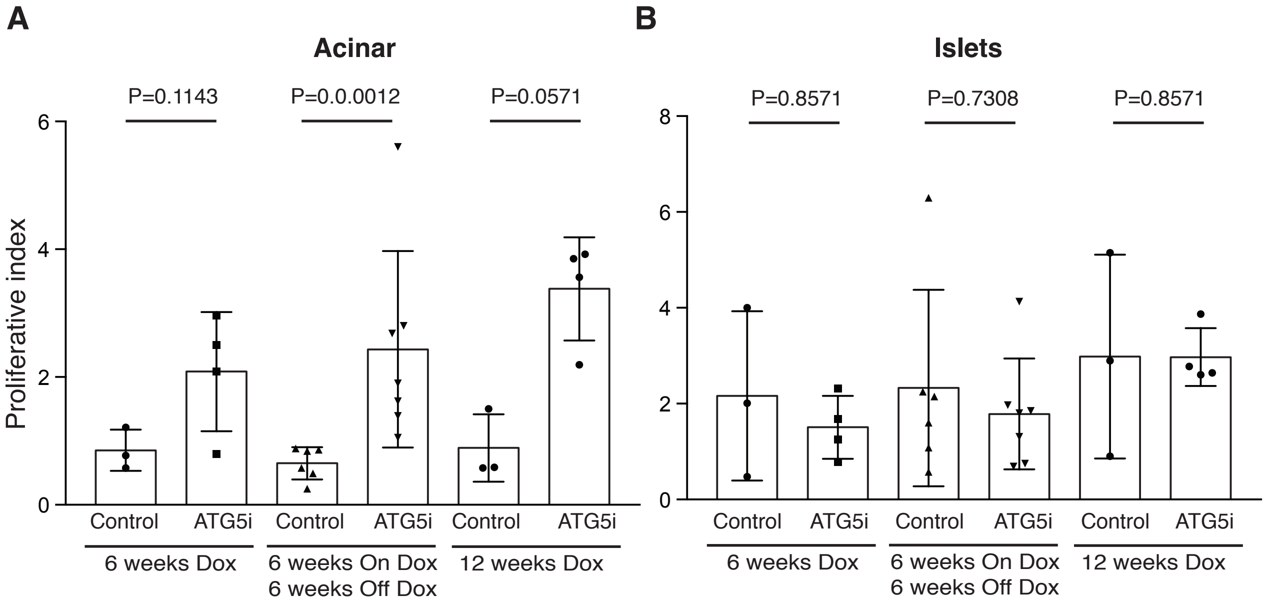

Figure S8. Proliferative index in the pancreas of ATG5i mice. (**A**) The pancreatic acinar of ATG5i mice displays a higher leveler of proliferation, as measured through the presence of MKI67-positive cells in IHC, in comparison to controls both during dox administration and even after dox was removed from the diet, although only the 6-weeks on dox and 6-weeks off dox regimen reaches significance. (**B**) Conversely the pancreatic islets did not show an increase in proliferation. Pairwise comparisons based on Mann-Whitney test.

Cassidy_Figure S9

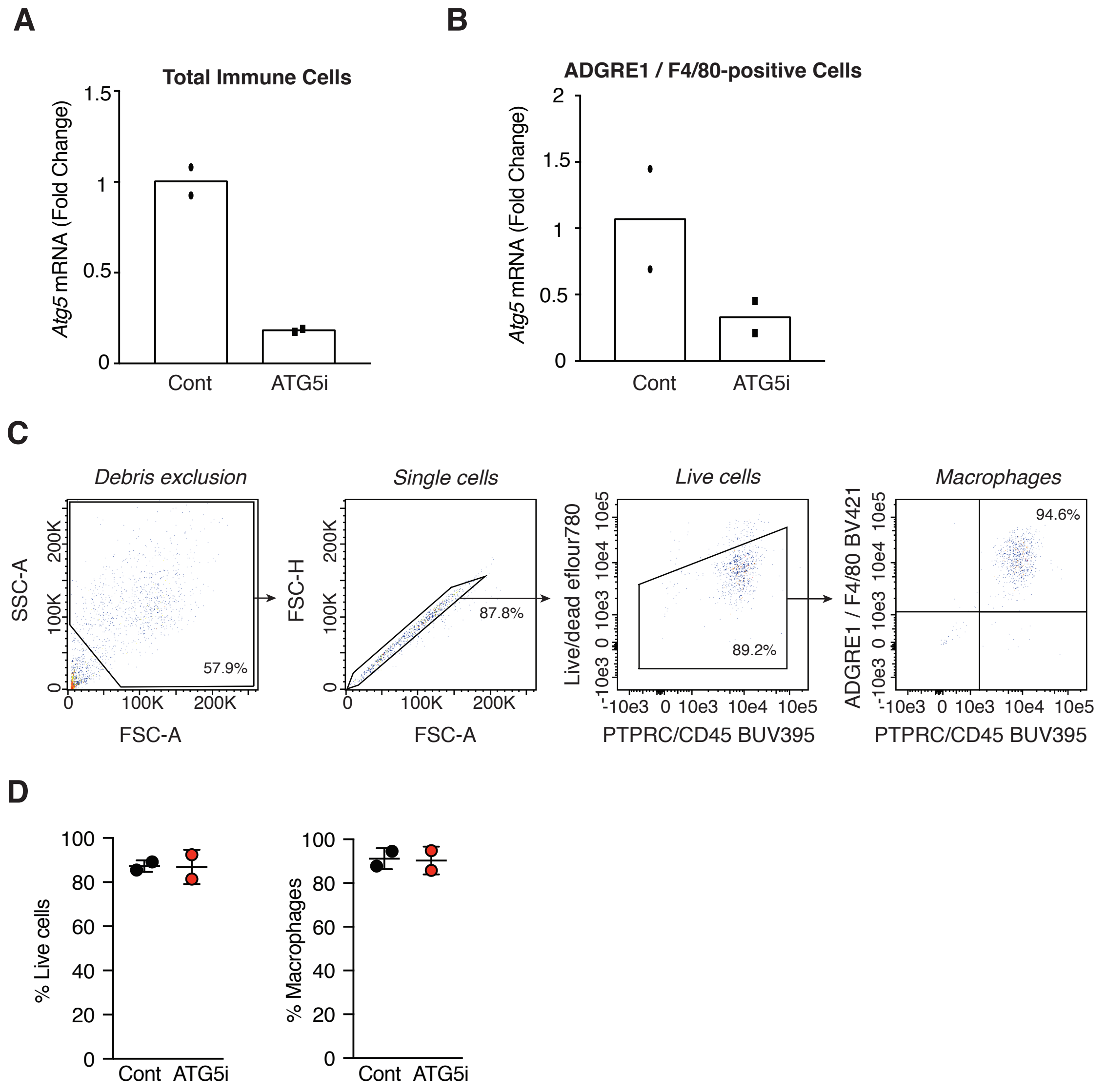

Figure S9. Immune cells residing in the liver of ATG5i mice display evidence of *Atg5* knockdown (**A-B**) qPCR for *Atg5* mRNA (relative to *Actb*) in total immune cells and macrophages (ADGRE1/F4/80-positive cells) isolated from control (cont) and ATG5i mice on dox for 6 weeks. (**C**) Example of flow cytometric analysis confirming a high level of macrophage enrichment from liver tissues after MACS purification using ADGRE1/F4/80 microbeads. FSC-A and FSC-H: area and height of forward scatter signal, respectively. SSC-A: area of side scatter signal. Brilliant Violet (BV) 421 and Brilliant Ultraviolet (BUV) 395 are fluorochromes to which anti-ADGRE1/F4/80 (macrophage marker) and anti-PTPRC/CD45 (general leukocyte marker) antibodies are conjugated, respectively. Live/dead eflour 780: far red Fixable Viability Dye. Values are arbitrary units. (**D**) Viability and purity of isolated macrophages.
